# Device-measured physical activity and cardiovascular disease risk in adolescent childhood cancer survivors. A physical activity in childhood cancer survivors (PACCS) study

**DOI:** 10.3389/fped.2022.977365

**Published:** 2022-08-25

**Authors:** Mari Bratteteig, Sigmund Alfred Anderssen, Corina Silvia Rueegg, Ellen Ruud, Ingrid Kristin Torsvik, Susi Kriemler, May Grydeland

**Affiliations:** ^1^Department of Sports Medicine, Norwegian School of Sport Sciences, Oslo, Norway; ^2^Oslo Centre for Biostatistics and Epidemiology, Oslo University Hospital, Oslo, Norway; ^3^Division for Pediatric and Adolescent Medicine, Department of Paediatric Haematology and Oncology, Oslo University Hospital, Oslo, Norway; ^4^Faculty of Medicine, Institute for Clinical Medicine, University of Oslo, Oslo, Norway; ^5^Department of Pediatrics, Haukeland University Hospital, Bergen, Norway; ^6^Epidemiology, Biostatistics and Prevention Institute, University of Zurich, Zurich, Switzerland; ^7^Department of Physical Performance, Norwegian School of Sport Sciences, Oslo, Norway

**Keywords:** cardiovascular disease risk, cardiometabolic risk, physical activity, accelerometry, childhood cancer survivors

## Abstract

**Objectives:**

We aimed to compare cardiovascular disease (CVD) risk factors in childhood cancer survivors (CCS) with age- and sex-stratified reference material and examine the association between physical activity (PA) intensities and CVD risk factors in CCS.

**Materials and methods:**

Within the cross-sectional, multicenter *Physical Activity in Childhood Cancer Survivors* (PACCS) study, we collected data on CVD risk factors [VO_2–*peak*_ (mL⋅kg^–1^⋅min^–1^), body mass index (BMI, kg/m^2^), systolic blood pressure (SBP, mmHg), and total-cholesterol/HDL-cholesterol (Total/HDL)] among CCS aged 9–18 years. CVD risk factors were compared to references with immediate *t*-tests. We transformed CVD risk factors into *z*-scores based on international references and generated an individual CVD risk score: [inverse ZVO_2–*peak*_ + Z_*BMI*_ + Z_*SBP*_ + Z_*Total/HDL*_)/4]. Multivariable mixed linear regression models were used to analyze the associations between device-measured PA intensities and CVD risk factors.

**Results:**

We included 157 CCS aged on average 13.4 years at inclusion and 8.2 years from diagnosis. Male CCS had lower VO_2–*peak*_ compared to references (45.4 vs. 49.4 mL⋅kg^–1^⋅min^–1^, *P* = 0.001), higher diastolic BP (67 vs. 63 mmHg, *P* < 0.001), lower HDL (1.35 vs. 1.44 mmol/L, *P* = 0.012), as well as a tendency to higher CVD risk score (*z*-score=0.14 vs. 0.00, *P* = .075). Female CCS’ CVD risk factors were comparable to references. Vigorous-intensity PA (VPA) was associated with CVD risk factors. A 10-min increase in VPA was associated with higher VO_2–*peak*_ (β = 4.9, 95% CI, 2.1–7.7), lower Total/HDL (β = −0.3, 95% CI, −0.6 to −0.1) and a lower CVD risk score (β = −0.4, 95% CI, −0.6 to −0.2).

**Conclusion:**

Male adolescent CCS had less favorable values of CVD risk factors compared to references. VPA in adolescent CCS is associated with clinically meaningful favorable values of CVD risk factors.

## Introduction

Due to major improvements in childhood cancer management, 5-year survival rates have increased to > 80% for children and adolescents diagnosed after the millennium (1–3). However, survival comes at a cost; due to intensive treatment during development and growth, childhood cancer survivors (CCS) are at particularly high risk of developing disease and treatment-related late effects that can interfere with physical and mental health, social functioning, and quality of life (4–6). Cardiovascular disease (CVD) risk factors, such as low cardiorespiratory fitness (CRF), adiposity, and abnormal glucose and lipid metabolism are important late effects among CCS associated with premature mortality in adulthood (7). Notably, CCS may face a sevenfold increased risk of cardiac mortality 30 years after diagnosis compared to age- and sex-matched references (8).

In children and adolescents with no history of cancer, physical activity (PA) is favorably associated with single and clustering of CVD risk factors (9–11). The same beneficial effects of PA on CVD risk is seen among adult CCS, and is therefore proposed as a strategy for secondary prevention and treatment (12–14). However, the relationship between PA and CVD risk in young CCS has not yet been thoroughly investigated, and existing studies are limited by small sample sizes and/or subjective measurement methods (15–21). Subjective measurement methods, such as questionnaires, are prone to measurement errors due to biases such as recognition-, memory- and social desirability (22), and have shown to be unreliable in pediatric populations (23). Existing studies suggest that there is an association between PA and body composition in adolescent CCS also. However, associations with other CVD risk factors remain uncertain. The objectives of this study were thus to compare CVD risk factors in adolescent CCS with age- and sex-stratified references and examine the association between device-measured PA intensities and CVD risk in adolescent CCS. We hypothesized that higher volume and higher intensity of PA are associated with a favorable CVD risk profile in CCS.

## Materials and methods

### Study design

This study is part of the international, cross-sectional, multicenter study *Physical Activity in Childhood Cancer Survivors (PACCS)* (24). The PACCS study consists of four work packages (WPs). The current study is based on WP2, which recruited CCS from WP1 from three study sites: Oslo University Hospital, Norway; Haukeland University Hospital, Norway; and University Children’s Hospital Basel, Switzerland. Manuals of procedures were developed to ensure standardized data collection across study sites. Participant recruitment and data collection were performed from January 2019 to December 2020.

### Participants

Childhood cancer survivors were recruited at their pediatric out-patient clinics when visiting for scheduled follow-up care. Inclusion criteria for the current study (WP2) were participation in WP1, age between 9–18 years, ability to perform a cardio-pulmonary exercise test (CPET), and cancer treatment completed ≥ 1 year prior to recruitment. Participants were excluded if they had language or cognitive difficulties, or a CPET was considered not possible due to physical or cognitive impairments.

We used reference values obtained by Stavnsbo et al. in 2018 as reference material (25). The material includes 5,084 females and 5,133 males aged 6–18 years and we used age- and sex-stratified reference values of the 9–18-year-olds (*n* = 5161–9229 in females and *n* = 5214–9214 in males, depending on the CVD risk factor).

### Outcomes: Single cardiovascular disease risk factors and cardiovascular disease risk score

Cardiorespiratory fitness was measured as VO_2–peak_ (mL⋅kg^–1^⋅min^–1^) by CPET. Gas exchange was determined by breath-by-breath sampling, averaged over 30-s intervals, through a breathing mask (Hans Rudolph Inc., 2700 series, Kansas City, MO, United States), and VO_2–peak_ was defined as the highest oxygen uptake during the test and was standardized for body mass. Criteria for aborting the CPET were decreasing systolic blood pressure (SBP) or multiple ventricular extrasystoles during the test. The CPET equipment was volume- and gas calibrated daily to ensure valid measurements, and the tests were performed by a physiotherapist in Bergen and exercise physiologists in Oslo and Basel according to standardized procedures.

In Oslo and Bergen, the CPET was performed by walking and running on a stationary treadmill (Rodby RL2700E, Vänge, Sweden, in Oslo; and Woodway PPS 55 Med, Woodway GmbH, Weil am Rhein, Germany, in Bergen). The breathing mask was connected to a metabolic analyzer (Jaeger Oxycon Pro, Viasys Healthcare GmbH, Hoechberg, Germany, in Oslo; and Jaeger Vyntus CPX, Vyaire Medical GmbH, Hoechberg, Germany, in Bergen). A modified Balke protocol for children was applied (26). Initial workload after habituation to the treadmill was 3 km/h, 4 km/h, and additionally 4% inclination, respectively, for the first 3 min. Thereafter, workload was increased every minute by increasing speed by 1 km/h and inclination by 2% every other minute, respectively. The test was stopped and considered maximal when the participant refused further increase in workload or until subjective exhaustion.

In Basel, the Godfrey cycling protocol was performed using an electronically braked ergometer (Ergoline 800; Pilger, St. Gallen, Switzerland) and a Quark B2 metabolic cart (Cortex MetaLyzer 3B, Leipzig, Germany). Work rate was increased every minute by 15–20 W, depending on participant’s height and physical fitness until the minimal cadence of 60 revolutions per minute could not be maintained or subjective exhaustion (27). The CPET protocol was different in Basel due to difference in equipment availability.

Reference values of VO_2–peak_ were also a combination of treadmill and ergometer cycle tests, where a correction factor of 1.05 was applied for children and adolescents who performed their VO_2–peak_ test on an ergometer cycle (28). In the current study, VO_2–peak_ values for participants from Basel were adjusted accordingly.

Systolic and diastolic blood pressure (DBP, mmHg) were measured with an electronic monitor in a seated position after a 5-min rest. Two measurements were performed, and the lowest value was registered.

Body mass was measured non-fasted and in light clothing to the nearest 0.1 kg by a digital scale. Height was measured to the nearest 1 mm by a stadiometer. Body mass index (BMI, kg/m^2^) was calculated.

Lipid metabolism was measured as cholesterol [Total-c (mmol/L), high-density lipoprotein cholesterol (HDL-c, mmol/L), ratio between Total-c and HDL-c (Total/HDL), and low-density lipoprotein cholesterol (LDL-c, mmol/L)]. Blood samples were collected in non-fasted state by venous sample in Oslo and Bergen, and by venous or capillary sample in Basel. Samples were analyzed by photometric methods at medical laboratories.

We generated *z*-scores for all of the above-mentioned single CVD risk factors based on the reference material and followed their guideline published in their Supplementary material (25). We first calculated age- and sex-specific reference values based on the published intercepts and beta-coefficients (25). Those reference values were then used to create *z*-scores based on the following formula: *z*-score = (mean_ccs_–mean_reference_)/SD_reference_. Stavnsbo et al. suggest using (natural) log-transformed values of BMI and Total/HDL to calculate the age- and sex-specific reference values, we thus log-transformed those two variables in our material accordingly. The resulting *z*-scores were then back-transformed to the original unit of 1 SD for meaningful interpretation (29).

Finally, we calculated a mean continuous CVD risk score = (inverseZ_*VO*2–peak_ + Z_BMI_ + Z_SBP_ + Z_Total/HDL_)/4. The CVD risk score was set to missing for participants with < 3 CVD risk factors (*n* = 1), and were thus omitted from analyses concerning CVD risk score. We calculated a CVD risk score as multiple CVD risk factors have shown to exert a synergetic effect on morbidity and mortality from CVD in later life, as compared to single risk factors (30, 31).

### Exposure: Physical activity intensities

A hip-worn accelerometer (ActiGraph GT3X-BT, Pensacola, FL, United States) was used to measure PA. Participants were instructed to wear the monitor for 8 consecutive days and to remove the monitor only for sleep and water-based activities. The accelerometers were initialized at a sampling rate of 30 Hz, and raw files were analyzed at 10-s epoch using the KineSoft analytical software version 3.3.80 (Loughborough, United Kingdom), restricted to hours between 06:00–23:59. Non-wear time was defined as periods of ≥ 20 consecutive minutes of zero counts. Minimum wear time of 8 h/day was required for a valid day, and ≥ 3 valid days were required for a person to be included in the analyses (Supplementary Figure 1). PA in the number of the participant’s valid days was averaged to represent their daily PA. Cut-points derived from counts per minute (cpm) were used to categorize the accelerometry data into light-intensity PA (LPA, 100–1999 cpm), moderate-intensity PA (MPA, 2000–5999 cpm), vigorous-intensity PA (VPA, ≥ 6000 cpm), and moderate-to-vigorous-intensity PA (MVPA, ≥ 2000 cpm), respectively (32–34).

### Covariates: Age, sex, puberty stage, parental education, and cancer-related characteristics

Puberty stage was determined by the self-reported Pubertal Development Scale questionnaire assessing indices of pubic hair, voice, and facial hair in males; and pubic hair, breast development, and menstruation in females (35). Participants were categorized as pre-pubertal if the participant reported the lowest category for all indices, post-pubertal if the participant reported the highest category for all indices, whereas the remaining participants were categorized as pubertal. Conversion of the original continuous Pubertal Development Scale into a 3-point ordinal scale is shown to be a reliable and valid tool in the pediatric population (36), albeit we are not aware of any validation studies performed in adolescent CCS.

Six categories for parent-reported parental education were collapsed into three categories: (1) 9–10 years; (2) 11–13 years; and (3) > 13 years.

Cancer diagnosis, and a limited number of available key factors from cancer treatment, that were available in conjunction with recruitment of participants, were extracted from medical records: cumulative anthracycline dose (Doxorubicin isotoxic equivalent dose, mg/m^2^) (37), cumulative radiation dose (Gy), and high-dose steroids (yes/no) as part of the cancer treatment protocol. Age at diagnosis and time since diagnosis were calculated.

### Statistical analyses

Characteristics of participants are expressed as mean ± SD or frequency (proportion), overall and stratified by sex. Comparisons between male and female CCS were made by Welch’s *t*-test for unequal variances for continuous variables, and by Chi square tests for categorical variables.

Comparison of CVD risk factors between CCS and references was performed using immediate *t*-tests with unequal variances. Associations between PA intensities and CVD risk factors were assessed using mixed effects linear regression models with study site as random intercept to account for clusters in the data. To adjust for potential confounding, we added covariates to the model (fixed effects) based on a directed acyclic graph drawn in Dagitty version 3.0^1^ (38) (Supplementary Figure 2). The following “minimal sufficient adjustment set” was identified: age, sex, puberty stage, parental education, age at diagnosis, time since diagnosis, and cancer treatment.

In the crude model, we adjusted only for the cluster variable study site (Model 1). In multivariable models, we additionally adjusted for age, sex, puberty stage and parental education (Model 2), and cancer-related variables (age at diagnosis, cumulative anthracycline dose, cumulative radiation dose, high-dose steroid treatment; Model 3). Time since diagnosis was omitted in Model 3 due to collinearity with age and age at diagnosis. We performed likelihood-ratio tests (LRT) to compare Model 2 and 3 in order to investigate influence of cancer-related characteristics on the PA-CVD risk factor associations. To compare models with LRT, *n* needs to be identical. Thus, missing parental education was defined as own category in the model and missing information on anthracycline dose (*n* = 9) and radiation dose (*n* = 1) was set to zero, avoiding loosing participants in analyses due to missing information on covariates. All *P*-values were two-sided, and we considered *P*-values ≤ 0.05 as statistically significant. Analyses were conducted using Stata statistical software release 16.0 (StataCorp LP, College Station, TX, United States).

### Ethics

*Physical Activity in Childhood Cancer Survivors* WP2 was approved by the Norwegian Regional Committee for Medical Research Ethics (project ID 2018/739), the Data protection Officer at Oslo University Hospital, and the Ethics Committee of North-Western and Central Switzerland (project ID 2019-00410). Written informed consent to participate in the current study was collected from all participants/parents.

## Results

### Study population

**FIGURE 1:**
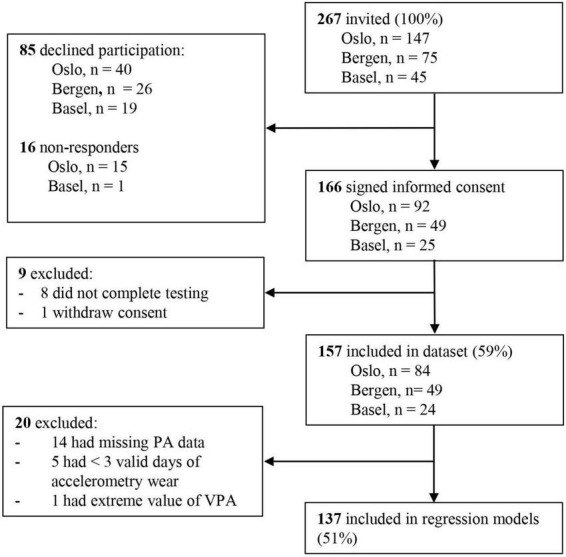
Flow chart of the inclusion process in PACCS WP2.

Of the 267 eligible invited CCS, 157 (59%) agreed to participate in the study and were included in descriptive analyses; 137 (51%) participants were included in the regression models (Figure 1).

The participants were on average 13.4 years old at inclusion and 8.2 years from diagnosis (Table 1). Half of the participants were survivors of leukemia, 78% had received anthracyclines, 29% had received radiotherapy, and 57% had received high-dose steroid treatment as part of their cancer treatment protocol. Females and males were comparable with respect to demographic and cancer-related characteristics, except borderline differences in distribution of puberty stage (18, 73, and 10% of the females were pre-pubertal, pubertal, and post-pubertal, respectively, whereas 27, 70, and 2% of the males were pre-pubertal, pubertal, and post-pubertal, respectively, *P*_trend_ = 0.077) and proportion experiencing relapse (4% in females vs. 12% in males, *P* = 0.077).

**TABLE 1:** Demographic and cancer-related characteristics in adolescent CCS, overall and stratified by sex.

	All (*n* = 157)	Females (*n* = 73)	Males (*n* = 84)	*P*
**Demographic characteristics**				
Age at study, years	13.4 ± 2.5	13.2 ± 2.7	13.5 ± 2.4	0.53
Puberty stage				0.077
Pre-pubertal	36 (23)	13 (18)	23 (27)	
Pubertal	112 (71)	53 (73)	59 (70)	
Post-pubertal	9 (6)	7 (10)	2 (2)	
Caucasian ethnicity	146 (93)	68 (93)	78 (93)	0.28
Parental education^a^				0.40
Primary school	6 (6)	3 (8)	3 (5)	
High school	32 (34)	9 (25)	23 (39)	
University or college	57 (60)	24 (67)	33 (56)	
**Cancer-related characteristics**				
Age at diagnosis, years	5.2 ± 3.4	5.4 ± 3.3	5.1 ± 3.4	0.59
Time since diagnosis, years	8.2 ± 3.6	7.9 ± 3.5	8.4 ± 3.6	0.34
Diagnoses (ICCC-3)				0.25
I Leukemias	78 (50)	39 (53)	39 (46)	
II Lymphoma	16 (10)	4 (5)	12 (14)	
III CNS tumors	18 (11)	7 (10)	11 (13)	
IV–XII other tumors	45 (29)	23 (32)	22 (26)	
Relapse	13 (8)	3 (4)	10 (12)	0.077
Anthracyclines	121 (78)	57 (79)	64 (77)	0.85
Cumulative dose (mg/m^2^) (31),^b^ (range)	161 ± 90 (45–450)	161 ± 91 (80–450)	161 ± 89 (45–410)	0.82
Radiotherapy	45 (29)	21 (29)	24 (29)	0.98
Cumulative dose (Gy) (range)^c^	33 ± 18 (12–70)	34 ± 20 (12–70)	32 ± 16 (12–54)	0.98
High-dose steroids^d^	90 (57)	42 (58)	48 (57)	0.96

Continuous variables are displayed as mean and standard deviation, categorical variables as frequency and proportion. There are no missing values besides the ones stated in the footnote below. CCS, childhood cancer survivors; CNS, central nervous system; CVD, cardiovascular disease; ICCC-3, International Classification of Childhood Cancer – third edition.

^a^Missing information on parental education for 62 participants.

^b^Missing cumulative anthracycline dose for nine participants.

^c^Missing cumulative radiation dose for one participant.

^d^As part of cancer treatment protocol (yes/no).

Basic characteristics were not significantly different between participants and non-participants in WP2 (Supplementary Tables 1, 2), though there was a tendency that fewer participants had experienced relapse compared to non-participants (9 vs. 17%, *P* = 0.10), and female participants were slightly younger than female non-participants (12.1 vs. 12.9 years, *P* = 0.11).

### Cardiovascular disease risk factors

Male CCS had lower VO_2–peak_ compared to references [45.4 vs. 49.4 mL⋅kg^–1^⋅min^–1^, *P* = 0.001 (Table 2)], as well as higher DBP (67 vs. 63 mmHg, *P* < 0.001) and lower HDL-c (1.35 vs. 1.44 mmol/L, *P* = 0.012). There were no differences between female CCS and references in any of the CVD risk factors.

**TABLE 2:** Comparison of single CVD risk factors in CCS vs. references, stratified by sex.

	Females			Males		
		
CVD risk factors	CCS (*n* = 73)^a^	References (25) (*n* = 5161)^b^	*P*-value	CCS (*n* = 84)^a^	References (25) (*n* = 5214)^b^	*P-value*
VO_2–peak_ (mL⋅kg^–1^⋅min^–1^)	40.1 ± 7.8	41.1 ± 6.6	0.25	45.4 ± 10.8	49.4 ± 7.7	0.001
BMI (kg/m^2^)	19.9 ± 3.8	20.0 ± 4.1	0.79	19.8 ± 3.6	20.0 ± 4.0	0.74
ln BMI	2.98 ± 0.18	2.98 ± 0.18	0.99	2.97 ± 0.17	2.97 ± 0.17	0.94
SBP (mmHg)	104 ± 10	105 ± 9	0.55	109 ± 9	109 ± 9	0.75
DBP (mmHg)	63 ± 9	63 ± 8	0.83	67 ± 9	63 ± 8	< 0.001
Total-c (mmol/L)	4.2 ± 0.8	4.3 ± 0.7	0.64	4.0 ± 0.8	4.1 ± 0.7	0.73
HDL-c (mmol/L)	1.47 ± 0.29	1.50 ± 0.32	0.30	1.35 ± 0.30	1.44 ± 0.32	0.012
Total/HDL	3.0 ± 0.8	3.0 ± 0.8	0.75	3.1 ± 0.9	3.0 ± 0.8	0.16
ln Total/HDL	1.06 ± 0.23	1.05 ± 0.24	0.64	1.10 ± 0.28	1.06 ± 0.25	0.15
LDL-c (mmol/L)	2.5 ± 0.8	2.4 ± 0.7	0.058	2.4 ± 0.7	2.2 ± 0.6	0.14

Variables are displayed as mean and standard deviation. BMI, body mass index; CCS, childhood cancer survivors; CVD, cardiovascular disease; DBP, diastolic blood pressure; HDL-c, high-density lipoprotein-cholesterol; LDL-c, low-density lipoprotein-cholesterol; ln, natural logarithm; SBP, systolic blood pressure; Total-c, total-cholesterol; VO2-peak, peak oxygen consumption.

^a^CVD risk factors varied from *n* = 72–73 in female CCS and *n* = 77–84 in male CCS.

^b^CVD risk factors varied from *n* = 5161–9229 in female references and *n* = 5214–9214 in male references.

**FIGURE 2:**
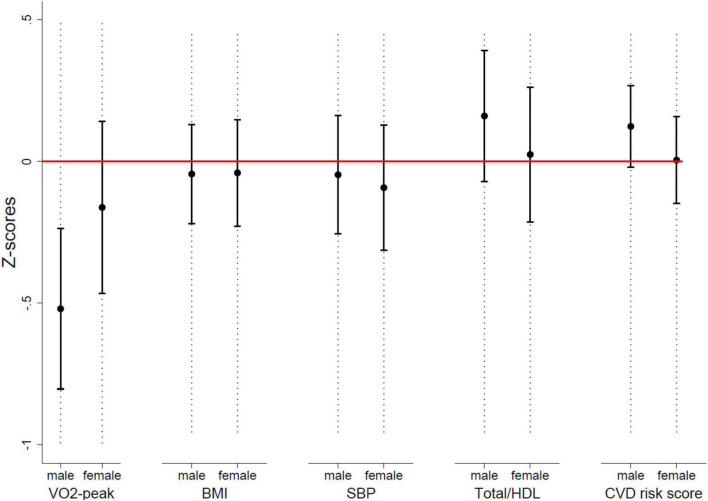
Mean *Z*-scores with 95% CI for single CVD risk factors and the CVD risk score, stratified by sex. BMI, body mass index (kg/m^2^); CVD, cardiovascular disease; SBP, systolic blood pressure; Total/HDL, total-cholesterol/HDL-cholesterol; VO2-peak, peak oxygen consumption.

Males had a *z*-score of −0.52 (95% CI, −0.82, −0.22, *P* = 0.001) for VO_2–peak_ and 0.14 (95% CI, −0.01, 0.30, *P* = 0.075) for the CVD risk score (Figure 2). Females had a *z*-score of −0.16 (95% CI, −0.45, 0.12, *P* = 0.26) for VO_2–peak_ and 0.02 (95% CI, −0.14, 0.18, *P* = 0.81) for the CVD risk score.

### Physical activity

In this substudy of PACCS, participants wore their accelerometer, on average, 13 h/day for 6 days. Participants engaged on average in 60 ± 28 min of MVPA/day (Table 3).

**TABLE 3:** Physical activity intensities in adolescent CCS, overall and stratified by sex.

	All (*n* = 137)	Females (*n* = 63)	Males (*n* = 74)
**Physical activity**			
LPA (min/day)	175 ± 49	172 ± 41	177 ± 55
MPA (min/day)	55 ± 25	52 ± 20	57 ± 29
VPA (min/day)	5 ± 5	5 ± 5	5 ± 5
MVPA (min/day)	60 ± 28	57 ± 23	62 ± 32

Variables are displayed as mean and standard deviation. CCS, childhood cancer survivors; LPA, low-intensity physical activity; MPA, moderate-intensity physical activity; MVPA, moderate-to-vigorous-intensity physical activity; PA, physical activity; VPA, vigorous-intensity physical activity.

### Associations between physical activity and cardiovascular disease risk factors

The fully adjusted model (Model 3) showed that all PA intensities were associated with VO_2–peak_, and the coefficients increased in size with higher intensity PA (Table 4). A 10-min increase in LPA, MPA, and VPA was associated with a higher VO_2–peak_ of 0.5 (95% CI, 0.1–0.8, *P* = 0.008), 1.0 (95% CI, 0.4–1.6, *P* = 0.001), and 4.9 mL⋅kg^–1^⋅min^–1^ (95% CI, 2.1–7.7, *P* = 0.001), respectively. Adding cancer-related variables to the model assessing the association between LPA and VO_2–peak_ did not alter the strength of the association. However, including cancer-related variables to the model resulted in a significantly better model fit (P_LRT_ comparing models = 0.002). Including cancer-related variables reduced the strength of the association between MPA and VO_2–peak_ from 1.3 to 1.0 mL⋅kg^–1^⋅min^–1^; and from 5.6 to 4.9 mL⋅kg^–1^⋅min^–1^ for the association between VPA and VO_2–peak_ (P_LRT_ comparing models = 0.011 and 0.004 for MPA and VPA, respectively).

**TABLE 4:** Associations between 10-min increase in PA intensities and CVD risk factors in adolescent CCS.

	10 min LPA (*n* = 137)			10 min MPA (*n* = 137)			10 min VPA (*n* = 137)		
			
	Model 1	Model 2	Model 3	Model 1	Model 2	Model 3	Model 1	Model 2	Model 3
	**β -coefficients with 95% CIs**								
VO_2–peak_ (mL⋅kg^–1^⋅min^–1^)^a^	0.4 (0.0 to 0.7)	0.5 (0.1 to 0.8)	0.5 (0.1 to 0.8)	1.2 (0.6 to 1.8)	1.3 (0.7 to 1.9)	1.0 (0.4 to 1.6)	5.5 (2.5 to 8.6)	5.6 (2.6 to 8.5)	4.9 (2.1 to 7.7)
BMI (kg/m^2^)	−0.2 (−0.3 to −0.1)	−0.1 (−0.2 to 0.1)	−0.0 (−0.2 to 0.1)	−0.3 (−0.5 to −0.0)	−0.1 (−0.3 to 0.1)	−0.1 (−0.3 to 0.2)	−1.1 (−2.3 to 0.2)	−0.8 (−2.0 to 0.3)	−0.8 (−2.0 to 0.3)
SBP (mmHg)^a^	−0.5 (−0.8 to −0.1)	−0.1 (−0.5 to 0.3)	−0.1 (−0.5 to 0.2)	−1.0 (−1.6 to −0.3)	−0.8 (−1.4 to −0.2)	−0.9 (−1.5 to −0.3)	−2.2 (−5.7 to 1.2)	−1.8 (−5.0 to 1.3)	−2.0 (−5.1 to 1.2)
Total/HDL^b^	−0.0 (−0.1 to 0.0)	−0.0 (−0.0 to 0.0)	−0.0 (−0.0 to 0.0)	−0.1 (−0.1 to −0.0)	−0.1 (−0.1 to −0.0)	−0.1 (−0.1 to −0.0)	−0.4 (−0.7 to −0.1)	−0.3 (−0.6 to −0.1)	−0.3 (−0.6 to −0.1)
CVD risk score^c^	−0.0 (−0.0 to 0.0)	−0.0 (−0.1 to 0.0)	−0.0 (−0.1 to 0.0)	−0.1 (−0.1 to −0.0)	−0.1 (−0.1 to −0.0)	−0.1 (−0.1 to −0.0)	−0.4 (−0.6 to −0.1)	−0.4 (−0.6 to −0.2)	−0.4 (−0.6 to −0.2)

BMI, body mass index; CCS, childhood cancer survivors; CVD, cardiovascular disease; LPA, low-intensity physical activity; MPA, moderate-intensity physical activity; PA, physical activity; SBP, systolic blood pressure; Total/HDL, ratio of total-cholesterol and high-density lipoprotein-cholesterol; VO2-peak: peak oxygen consumption; VPA, vigorous-intensity physical activity. Adjustments: Model 1 is adjusted for site; Model 2 is additionally adjusted for age, sex, puberty stage, and parental education; Model 3 is additionally adjusted for age at diagnosis, cumulative anthracycline dose, radiation dose, and high-dose steroid treatment (yes/no).

^a^Missing information on VO2-peak (unknown reason) and SBP in two participants.

^b^Missing information on Total/HDL in six participants.

^c^CVD risk score was set to missing for one participant due to < 3 CVD risk factors.

Both MPA and VPA were associated with lower Total/HDL (−0.1, 95% CI, −0.1 to −0.0, *P* = 0.022; and −0.3, 95% CI, −0.6 to −0.1, *P* = 0.016, respectively), and MPA was additionally associated with lower SBP (-0.9 mmHg, 95% CI, −1.5 to −0.3, *P* = 0.005). Adding cancer-related variables to the model did not affect the association between PA intensities and Total/HDL or SBP. None of the PA intensities were associated with BMI and including cancer-related variables in the model did not affect the associations.

Also, MPA and VPA were associated with the CVD risk score. A 10-min increase in MPA and VPA were associated with a lower CVD risk score of −0.1 (95% CI, −0.1 to −0.0, *P* < 0.001), and −0.4 (95% CI, −0.6 to −0.2, *P* = 0.001), respectively. Adding cancer-related variables to the model did not affect the association between PA intensities and the CVD risk score.

## Discussion

### Main findings

We found that male adolescent CCS had lower VO_2–peak_ and HDL-c, and higher DBP and CVD risk score compared to references, in contrast to female adolescent CCS where all CVD risk factors were comparable to references. To our knowledge, this is the first study examining the association between device-measured PA and CVD risk factors in adolescent CCS. We found that PA was associated with single CVD risk factors and the CVD risk score.

### Comparison to other studies

#### Cardiorespiratory fitness

With respect to the single CVD risk factors, we found that PA at any intensity was positively associated with VO_2–peak_, and higher intensity PA was inversely associated with Total/HDL. MPA was additionally inversely associated with SBP. Our results are in line with a previous study by Jarvela et al. showing a positive association between self-reported PA and VO_2–peak_ in young adult survivors of acute lymphoblastic leukemia (15).

#### Adiposity

Our results conflict with previous studies by Slater et al. showing inverse associations between self-reported PA and adiposity in adolescent and young adult CCS (18, 19). These studies used waist circumference, body fat percentage, subcutaneous and visceral adipose tissue as measures of adiposity, and PA was assessed by questionnaire, which might explain the differences in results. This claim is supported by two other studies in CCS: Tonorezos et al. found an inverse association between device-measured PA and body fat percentage, but not with BMI, in young adult CCS (21); Jarvela et al. found that an increase in PA led to reduced adiposity, measured as waist circumference, waist-to-hip ratio, and body fat percentage, but not with BMI (15). Thus, BMI does not seem like an appropriate measure of adiposity in CCS to detect a potential association with PA. BMI does not distinguish between fat mass and fat-free mass, and studies in CCS have shown that despite having similar BMI *z*-score as healthy controls, they have deficits in fat-free mass and excesses of fat mass (39).

#### Blood pressure and lipid metabolism

Jarvela et al. additionally found that an increase in PA resulted in lower SBP and higher HDL-c (15). Our results are in line with these findings. We did not look at the association between measures of PA and HDL-c separately. However, we found an inverse association between PA and ratio of Total/HDL, which might be explained by higher HDL-c. Other cross-sectional studies in CCS have failed to detect these associations (18, 19, 21). This might be due to methodological differences in measuring PA.

#### Influence of cancer treatment on the association between physical activity and cardiovascular disease risk factors

A recent study by Schindera et al. found a strong association between CRF and CVD risk factors in young adult CCS (40). They report that the associations did not change noticeably when adjusting the analyses for cancer treatment. They suggested that survivors may have modified their CVD risk through CRF. Our statistical models were similar, where we compared the associations with- and without including treatment variables as confounders. We found that cancer-related variables reduced the strength of the association between PA and VO_2–*peak*,_ which might indicate that CCS have smaller increase in VO_2–peak_ in response to PA than adolescents with no history of cancer. Some potential mechanisms induced by cancer treatment, may be that cardiovascular diseases limit performance during exercise through impairments in systolic and diastolic function, or heart rate response; pulmonary limitations may cause impairments in ventilation and gas exchange; and arterial stiffness and endothelial dysfunction may limit the vascular system (41). We found no impact of cancer-related variables on the association between PA and the other CVD risk factors, suggesting that PA is associated with SBP and Total/HDL independently of former cancer treatment.

### Strengths and limitations

The strengths of this study are the inclusion of adolescent CCS, and the use of reliable and valid measurement methods, including device-measured PA and directly measured VO_2–peak_. We also had access to some key cancer-related characteristics, enabling us to adjust for them in the analyses. Moreover, we recruited participants with a history of various childhood cancer diagnoses. Most previous studies have included participants with a history of acute lymphoblastic leukemia, only. This study has also limitations to be considered. Our analyses assume directional associations, however, associations of cross-sectional data have no real direction. We were thus unable to draw any causal inference due to the cross-sectional design. We did not have fasted blood sample values as the references did. However, Total-c and HDL-c are mainly unaffected by fasted state (42). Moreover, the use of BMI as a measure of adiposity might have reduced the possibility to find an association between PA and adiposity. Self-reporting of puberty stage might be prone to bias, however, a recent study found substantial agreement between Pubertal Development Scale and Tanner stage when each scale was combined into three categories (36), as we did. We failed in finding reference material for HbA1c, and we were thus unable to compare a measure of glucose metabolism, which is a central CVD risk factor, in CCS to references. Lastly, the relatively low inclusion rate (59%) might reflect that an invitation to participate in a study with physical performance testing might appeal more to those who are regularly physically active and may thus have led to selection bias. Even though of no statistical difference, our analysis of non-participants showed that those who had experienced relapse, and females of higher age, were less likely to participate in the current study (WP2).

## Conclusion

We found that male adolescent CCS had less favorable values of CVD risk factors compared to references. Moreover, we found higher levels of PA to be associated with a more favorable CVD risk profile in adolescent CCS. This highlights the need to encourage and help adolescent CCS to increase or maintain their PA level. Whether higher CVD risk is due to lower PA level is yet to be determined. Randomized controlled trials or cohort studies are needed to explore whether increased PA can reduce CVD risk in adolescent CCS.

## Data availability statement

The original contributions presented in this study are included in the article/Supplementary material, further inquiries can be directed to the corresponding author.

## Ethics statement

The study was reviewed and approved by the Regional Committee for Medical and Health Research Ethics (2018/739). Written informed consent to participate in this study was provided by the participants or their legal guardian/next of kin.

## Author contributions

MB: formal analysis, investigation, methodology, visualization, writing original draft, and writing – review and editing. SA: conceptualization, funding acquisition, methodology, project administration, supervision, visualization, and writing – review and editing. CR: methodology, supervision, validation, visualization, and writing – review and editing. ER: conceptualization, funding acquisition, project administration, supervision, and writing – review and editing. IT and SK: investigation and writing – review and editing. MG: conceptualization, funding acquisition, investigation, methodology, project administration, supervision, and writing – review and editing. All authors contributed to the article and approved the submitted version.

## Footnotes

1
www.dagitty.net
