# Supplementary material for: Device-measured physical activity and cardiovascular disease risk in adolescent childhood cancer survivors. A physical activity in childhood cancer survivors (PACCS) study
Source: Front Pediatr. 2022 Aug 25;10:977365. doi: 10.3389/fped.2022.977365 (PMC9453306; doi:10.3389/fped.2022.977365)
Supplement: Supplementary file 1 [file Data_Sheet_1.pdf]

Supplementary Material

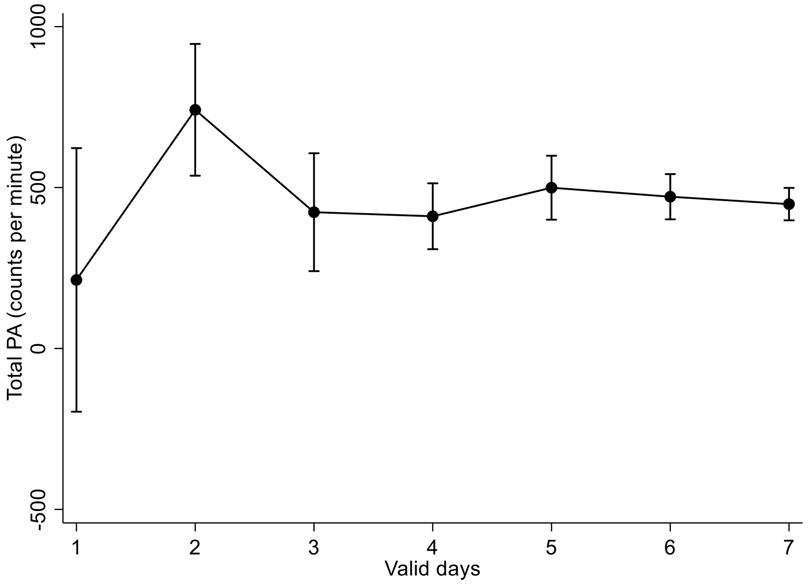

Figure 1: Margins plot showing participant's total physical activity (mean counts per minute/day with 95% CI) according to their respective number of valid days (n = 143)

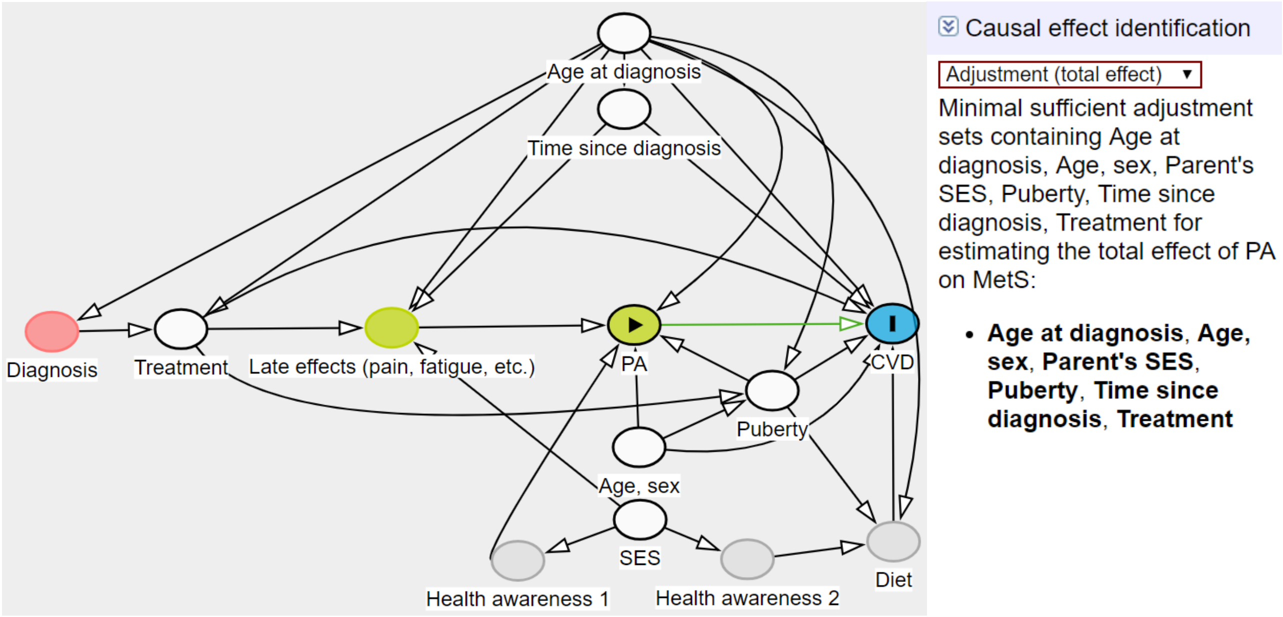

Figure 2: Directed acyclic graph created in *Dagitty*

Abbreviations: CVD, cardiovascular disease (risk factors); PA, physical activity; SES, socioeconomic status

Table 1: Key characteristics of non-participants and participants in PACCS WP2

	Non-participants (n = 89)[a]	Participants (n = 157)	P
Sex<br>Girls<br>Boys	 40 (45)<br>49 (55)	 73 (47)<br>84 (54)	.81
Age at inclusion in WP1, years[b]	12.4 ± 2.2	12.1 ± 2.2	.30
Age at diagnosis, years[c]	5.1 ± 3.1	5.1 ± 3.4	.93
Time since diagnosis in WP1, years[d]	7.1 ± 3.2	6.8 ± 3.2	.56
Diagnosis (ICCC-3)<br>I. Leukemia<br>II. Lymphoma<br>III. Tumor CNS<br>IV-XII Tumor other	 43 (48)<br>11 (12)<br>10 (11)<br>25 (28)	 78 (50)<br>16 (10)<br>18 (11)<br>45 (29)	.96
Relapse	15 (17)	14 (9)	.10

Note: Values are based on data extracted from medical records prior to recruitment to PACCS WP1. Continuous variables are displayed as mean and standard deviation, categorical variables as frequency and proportion. There are no missing values besides the ones stated in the footnote below.

a: Missing key characteristics of non-participants from Basel (n = 21).

b: Missing age at inclusion for 2 participants.

c: Missing age at diagnosis for 12 non-participants and 6 participants.

d: Missing time since diagnosis for 6 participants.

Table 2: Key characteristics of non-participants and participants in PACCS WP2, stratified by sex

	Females			Males		
	Non-participants (n = 40)[a]	Participants (n = 73)	P	Non-participants (n = 49)[a]	Participants (n = 84)	P
Age at inclusion in WP1, years[b]	12.9 ± 2.1	12.1 ± 2.4	.11	12.0 ± 2.1	12.1 ± 2.0	.94
Age at diagnosis, years[c]	4.8 ± 3.3	5.1 ± 3.2	.59	5.4 ± 3.0	5.1 ± 3.5	.68
Time since diagnosis in WP1, years[b]	7.9 ± 3.5	6.8 ± 3.2	.09	6.4 ± 2.8	6.8 ± 3.2	.37
Diagnosis (ICCC-3)<br>I. Leukemias<br>II. Lymphoma<br>III. CNS tumors<br>IV-XII Other tumors	 21 (53)<br>4 (10)<br>4 (10)<br>11 (28)	 39 (53)<br>4 (5)<br>7 (10)<br>23 (32)	.81	 22 (45)<br>7 (14)<br>6 (12)<br>14 (29)	 39 (46)<br>12 (14)<br>11 (13)<br>22 (26)	.99
Relapse	6 (15)	4 (5)	.16	9 (18)	10 (12)	.32

Note: Values are based on data extracted from medical records prior to recruitment to PACCS WP1. Continuous variables are displayed as mean and standard deviation, categorical variables as frequency and proportion. There are no missing values besides the ones stated in the footnote below.

a: Missing key characteristics of non-participants from Basel (n = 21).

b: Missing age at inclusion for 2 female participants.

c: Missing age at diagnosis for 4 female and 8 male non-participants and 5 female and 1 male participants.

d: Missing time since diagnosis for 5 female and 1 male participants.
